# Pathology after a combination of sequential and simultaneous unipolar radiofrequency ablation of ventricular tachycardia in a postmortem heart with cardiac sarcoidosis

**DOI:** 10.1002/ccr3.1577

**Published:** 2018-05-08

**Authors:** Koji Miyamoto, Taka‐aki Matsuyama, Takashi Noda, Hatsue Ishibashi‐Ueda, Kengo Kusano

**Affiliations:** ^1^ Department of Cardiovascular Medicine National Cerebral and Cardiovascular Center Osaka Japan; ^2^ Department of Pathology National Cerebral and Cardiovascular Center Osaka Japan

**Keywords:** ablation, nonischemic cardiomyopathy, pathology, ventricular tachycardia

## Abstract

This report shows a postmortem examination of a heart performed in a patient with cardiac sarcoidosis undergoing a sequential and simultaneous unipolar radiofrequency ablation. A combination of a sequential and simultaneous unipolar radiofrequency ablation might be useful for creating transmural ablation lesions on the interventricular septum in patients with cardiac sarcoidosis.

## 1 INTRODUCTION

A postmortem examination of the heart was performed in a patient with cardiac sarcoidosis undergoing simultaneous unipolar radiofrequency (SURF) ablation on the interventricular septum. The pathologic findings showed that a transmural lesion had been created with the SURF ablation.

Controlling ventricular tachycardias (VTs) is important in patients with nonischemic cardiomyopathy (NICM). A combined approach to the VT management using antiarrhythmic drugs and radiofrequency catheter ablation (RFA) is often required to control such VTs.^1^, ^2^, ^3^

Because the VTs in patients with NICM occur based on a complex underlying substrate in the endocardium, epicardium, and/or intramural region, transmural lesion creation is necessary to manage these VTs. Saline‐irrigated catheters create deeper and larger ablation lesions than nonirrigated catheters and improve the outcome of RFA.^4^ However, despite the use of open‐irrigated ablation catheters, creating transmural ablation lesions is difficult, and the management of VTs in patients with NICM is still challenging.^5^, ^6^, ^7^, ^8^, ^9^

To overcome the limited lesion creation with RFA, some techniques to improve it have been developed, such as simultaneous unipolar radiofrequency (SURF) ablation, bipolar ablation, needle ablation, and transcoronary ethanol ablation.^10^, ^11^, ^12^, ^13^, ^14^, ^15^, ^16^, ^17^ In this case report, we evaluated the pathologic features after a SURF ablation of a VT in a postmortem heart with cardiac sarcoidosis.

## 2 CASE

The patient was a 65‐year‐old man histopathlogically diagnosed with cardiac sarcoidosis at the age of 35 years. The 12‐lead electrocardiogram exhibited an intraventricular conduction disturbance and left superior axis. The left ventricle (LV) exhibited a progressive dilation with a contractile dysfunction (severe hypokinesis on the inferior and inferior and septum). He was implanted with a cardiac resynchronization therapy device with defibrillator capability. In spite of the administration of amiodarone 100 mg, sotalol 160 mg, carvedilol 7.5 mg, mexiletine 400 mg, enalapril 2.5 mg, and spironolactone 25 mg, the patient was admitted to our hospital due to repetitive drug‐resistant VT episodes and decompensated heart failure. The LV exhibited dilation and contractile dysfunction with an LV ejection fraction of 18%. In addition to the amiodarone, sotalol, carvedilol, mexiletine, enalapril, and spironolactone, intensive treatment of a VT storm and decompensated heart failure was performed with lidocaine 40‐100 mg/h and furosemide; however, the VTs still could not be controlled.

### 2.1 Electrophysiological examination and ablation procedure

**Figure 1:**
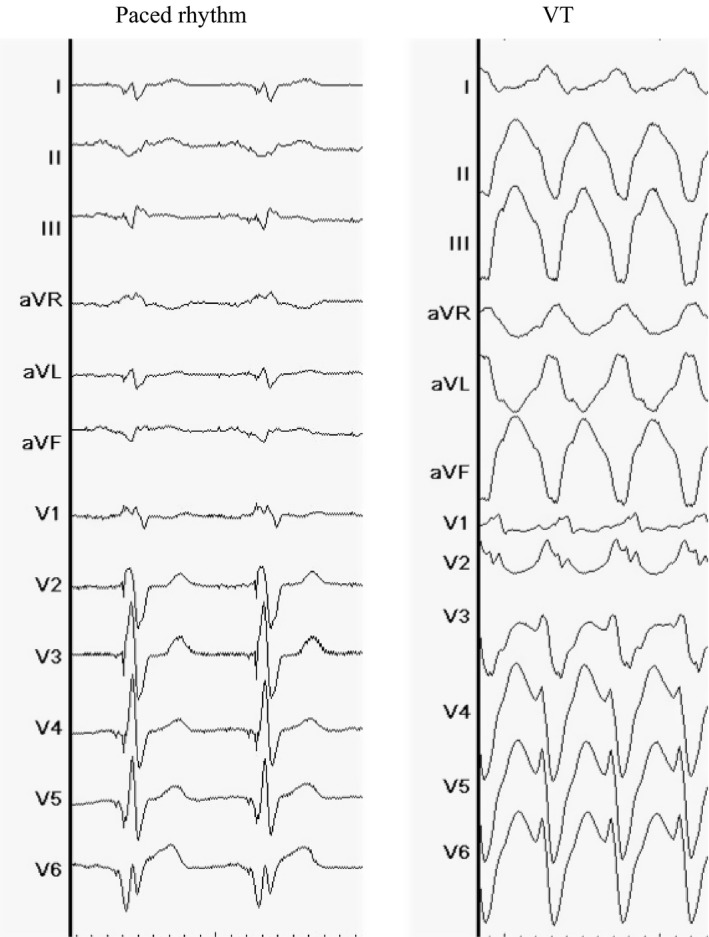
A 12‐lead Electrocardiogram at Baseline (Paced Rhythm) and the Clinical VT. The VT exhibited a right bundle branch block pattern and left superior axis. The VT cycle length is 380 ms. VT, ventricular tachycardia

The clinical VT on the 12‐lead electrocardiogram exhibited a right bundle branch block pattern and left superior axis (Figure 1). The VT cycle length was 380 millisecond, with a widened QRS duration of 184 millisecond. RFA was performed to manage the VT storm.

**Figure 2:**
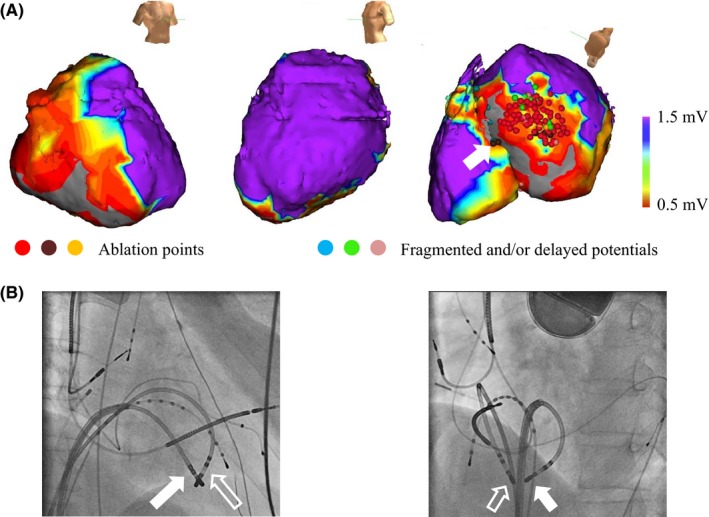
Voltage Map and Fluoroscopy at RFA. A, Bipolar voltage map at baseline of the LV and RV from the right anterior oblique, left lateral, and inferior projections. Low voltage zones defined as <1.5 mV were mainly located on the IVS and postero‐inferior wall of the LV. The red, brown, and yellow dots indicate the ablation points, and the blue, green, and pink dots indicate the abnormal electrocardiograms such as fragmented and/or delayed potentials. The white arrow indicates the SURF ablation site. B, Fluoroscopy shows the catheter position when performing the SURF ablation. The two electrodes on both sides of the inferior IVS were located at opposite sites. The open arrows indicate the ablation catheter on the IVS from the RV, and the closed arrows indicate the ablation catheter on the IVS from the LV. IVS, interventricular septum; LV, left ventricle; RFA, radiofrequency catheter ablation; RV, right ventricle; SURF, simultaneous unipolar radiofrequency ablation

Steerable catheters were inserted from the right femoral vein and placed in the right atrium and ventricle of interest. The LV endocardium was accessed using the trans‐septal approach. Electroanatomical mapping was performed with EnSite (Abbott, Chicago, IL). A 3.5‐mm open‐irrigated ablation catheter (Therapy^™^ Cool Path^™^ Duo: Abbott) was used for the ablation and mapping. Bipolar voltage maps of the endocardium of the LV and right ventricle (RV) were constructed at baseline (pacing rhythm). Low voltage zones, defined as <1.5 mV, were mainly located on the interventricular septum (IVS) and postero‐inferior wall of the LV. The clinical VT was induced and was hemodynamically stable. Entrainment mapping revealed that the VT had a reentrant mechanism. Activation mapping was performed; however, it could not depict the entire circuit, suggesting that a part of the circuit was intramural and/or epicardial. We performed RFA on the inferior and infero‐septal walls of the LV and IVS from both the RV and LV endocardium, based on the activation and/or substrate maps, targeting the low voltage zones and/or abnormal electrograms such as those with fragmented and double potentials (Figure 2A). The procedural endpoint was the noninducibility of the clinical VTs. RF current was delivered for up to 60 seconds in the power‐controlled mode with 30‐45 watt and an irrigation rate of 17 mL/min.

We performed sequential RFA from the LV and RV; however, the VT did not terminate or slow. A SURF ablation on the inferior IVS from both the LV and RV endocardium was also performed. The ablation catheter used on the LV was a 3.5‐mm open‐irrigated ablation catheter (Therapy^™^ Cool Path^™^ Duo: Abbott), and that on the RV was a 4‐mm nonirrigated ablation catheter (Therapy^™^ Thermocouple: Abbott). When performing the SURF ablation, two separate dispersive patches were used as the indifferent electrodes, with two separate generators in the power‐control mode for the delivery of the SURF. RF current was delivered in the power‐control mode starting at 10 watts, independently titrated up to 30 watts for each catheter with care taken to limit the temperature to <42°C for the irrigated catheter and <55°C for the nonirrigated catheter. The RF delivery was discontinued when the catheter tip impedance of either catheter dropped by more than 15 Ω as monitored from both RF generators. Figure 2B shows the position of the two ablation catheters when the SURF ablation was performed from both the RV and LV endocardium. We performed the SURF ablation at two sites on the IVS. The mean percentage of the R‐wave reduction achieved by the SURF ablation was 67% on the LV endocardium and 86% on the RV endocardium. The VT was terminated and/or slowed during the RF application on the inferior wall of the LV and inferior IVS. The total application time of the RFA was 3502 seconds.

The clinical VT could not be induced at the end of the procedure. However, a VT with a slightly different QRS morphology emerged 2 days after the session and his heart failure worsened and became more serious due to VT storms. He developed pulseless electrical activity after that, and required intubation, sedation with propofol, percutaneous cardiopulmonary support, and intra‐aortic balloon pumping. A second ablation session was performed 9 days after the first session. The VT was eliminated by RF applications on the infero‐septal wall of the LV. No further VTs emerged after the RFA. The patient, however, died from deterioration of his heart failure 12 days after the procedure. A postmortem examination of the heart was performed.

### 2.2 Postmortem pathologic findings

**Figure 3:**
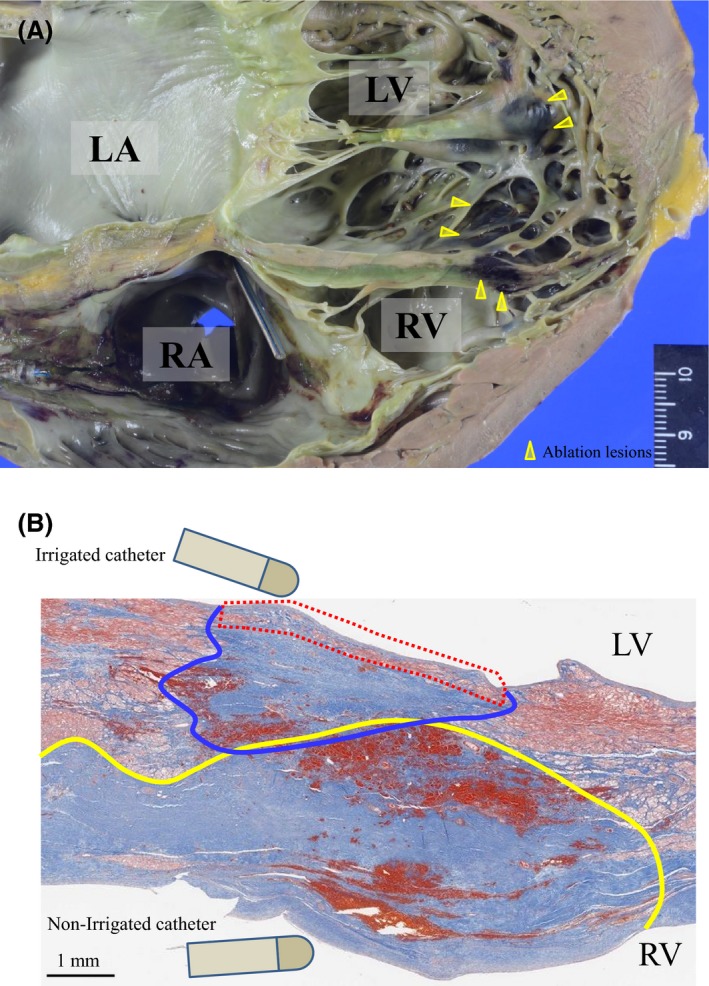
Macro‐ and Microscopic Findings at SURF Sites. A, Macroscopic overview of the heart at the level of the IVS in a four chamber slice. The yellow arrow heads indicate the ablation lesions, which are mainly located on the IVS and infero‐posterior wall of the LV. B, Microscopic findings at the SURF site on the IVS with Masson's trichrome stain. Irregular fibrotic layers that were originally caused by cardiac sarcoidosis, diffusely extend into the IVS. The area surrounded by the blue circle represents the ablation lesions created from the LV endocardium. The area surrounded by the yellow circle represents the ablation lesions created from the RV endocardium. Transmural ablation lesions could be created; however, there is a little spared visible myocardial tissue on the LV endocardial surface (surrounded by the red dotted line). IVS, interventricular septum; LV, left ventricle; RV, right ventricle; SURF, simultaneous unipolar radiofrequency ablation

Figure 3A shows a cross section of the ablation lesions on the IVS in a four chamber slice, where the ablation lesions from the SURF ablation were assessed. The heart was significantly enlarged and weighed 505 g. The ventricular septum was thin. The yellow arrow heads in Figure 3A indicate the ablation lesions. The black‐colored areas were lesions with hemorrhaging caused by damage to small intramural coronary arteries and capillaries and the adjacent cloudy discolored areas were necrotic tissue caused by the RF energy. The ablation lesions were mainly located on the IVS and inferior wall of the LV. Figure 3B shows identical histologic sections to those ablation lesions on the IVS in Figure 3A. The original pathologic fibrosis resulting from the cardiac sarcoidosis extended diffusely onto the IVS. In Figure 3B, the area surrounded by the blue line was assumed to be the ablation lesions created from the LV endocardium, and the area surrounded by the yellow line was the lesions created from the RV endocardium. Those lesions were contiguous, creating a transmural ablation lesion on the IVS. There was, however, a little spared viable myocardial tissue that escaped the RF energy on the LV endocardial surface (area surrounded by the red dotted line) despite the deeper sites having been completely ablated.

There was no active epithelioid granuloma from the sarcoidosis observed in the autopsy.

## 3 DISCUSSION

To the best of our knowledge, this case provided important information from the analysis of the pathologic changes after the SURF ablation with open‐irrigated and nonirrigated catheters for the first time in a human heart. We focused on the two major findings in this heart, as described below.

### 3.1 Efficacy and safety of the SURF

Iyer et al reported a case of incessant VT originating from the IVS, that could not be eliminated by a repeat sequential catheter ablation from both sides of the IVS; however, the VT was successfully controlled with a SURF ablation with two ablation catheters on both sides of the IVS.^17^ Yamada et al^16^ assessed the efficacy and safety of a SURF ablation in treating intramural LVOT VAs. They showed that a SURF ablation for VAs originating from the intramural LVOT could eliminate VAs without any complications in some cases that a sequential unipolar RF ablation failed from both endocardial and epicardial sites. The possible reasons for a greater efficacy of the SURF ablation compared with the usual unipolar ablation for VTs with intramural origins are:

1. An increased current density in the intramural region, leading to a rise in the tissue temperature in the region (resistive heating)
2. Increased conductive heating in the intramural region due to resistive heating from both sides of the wall.

There are some other approaches for creating transmural lesions, such as bipolar ablation, needle ablation, and transcoronary ethanol ablation.^10^, ^11^, ^12^, ^13^, ^14^, ^15^ Bipolar radiofrequency current was applied between two distal ablation catheter electrodes on both sides of the myocardium. A high effectiveness of a bipolar ablation has been reported; however, there are some disadvantages as compared to the SURF ablation.^12^, ^15^, ^18^ When performing a SURF ablation, we can monitor the biophysical parameters of the lesion creation, including the power delivery, local electrode temperature, and impedance profiles on both sides of the myocardium. On the other hand, we can only monitor these parameters on one side of the wall when performing a bipolar ablation. In addition, a SURF ablation allows for independent power and temperature control settings while monitoring the biophysical parameters. On the other hand, a bipolar ablation can only deliver the same RF current from both ablation electrodes. Therefore, a SURF ablation is thought to be more controllable and predictable in creating ablation lesions and is likely more efficient and safer than bipolar ablation.

### 3.2 Endocardial sparing after ablation using an open‐irrigated catheter

In this case, ablation in the LV was performed with an open‐irrigated catheter, and there was a little spared viable myocardial tissue that escaped the RF energy on the superficial LV endocardium. On the other hand, there was no residual viable myocardium on the RV endocardial surface, where the RF current was delivered with a nonirrigated catheter.

As the possible reason for the residual viable myocardium on the LV endocardial surface, Di Biase et al^19^ proposed the “endocardial sparing” phenomenon, defined as a lesion with the presence of nonablated endocardium when using an irrigated catheter despite the presence of deeper tissue damage. This phenomenon might be associated with endocardial surface cooling by an open‐irrigated catheter and/or movement of the catheter during the RF current delivery. The endocardial surface is often complicated in patients with NICM, whose endocardial trabeculae are compensatorily hypertrophied because of myocardial fibrosis. Endocardial sparing may easily occur in NICM patients with a complicated endocardium because the catheter contact with the endocardium is not stable.

### 3.3 Limitations

We performed RF ablation from one side on the RV and LV endocardium sequentially before the SURF ablation, and the postmortem pathologic findings in this report were not created only by the SURF ablation. Therefore, it would be reasonable to think that the transmural RF lesion was created by a combination of the sequential RF ablation and SURF ablation. There have been only a few clinical reports about the SURF ablation, and therefore the optimal ablation settings, including the power, RF delivery time, and type of ablation catheter, are unclear. The RF lesions seem to be greater on the RV side than the LV side in Figure 3. However, it seems to be greater on the LV side in another cross section (Figure S1). It is difficult to estimate and compare the entire ablation lesion volume. Further investigation with a larger patient population is required to examine that.

## 4 CONCLUSION

To the best of our knowledge, this is the first case showing the important pathologic information after a SURF ablation and a sequential RF ablation in a human heart. This pathological report revealed the efficacy of the SURF ablation and sequential RF ablation in creating transmural ablation lesions on the IVS.

## CONFLICT OF INTEREST

The authors have no conflict of interests to declare.

## AUTHORSHIP

KM and TM: involved in conception and design, analysis and interpretation of data, drafting of the manuscript and revising it critically, and final approval of the manuscript. TN, HI‐U, and KK: involved in analysis and interpretation of data, revising the manuscript critically, and final approval of the manuscript.
