# Supplementary figures and images for: Pathology after a combination of sequential and simultaneous unipolar radiofrequency ablation of ventricular tachycardia in a postmortem heart with cardiac sarcoidosis
Source: Clin Case Rep. 2018 May 8;6(7):1219–24. doi: 10.1002/ccr3.1577 (PMC6028406; doi:10.1002/ccr3.1577)

Supplemental Figure

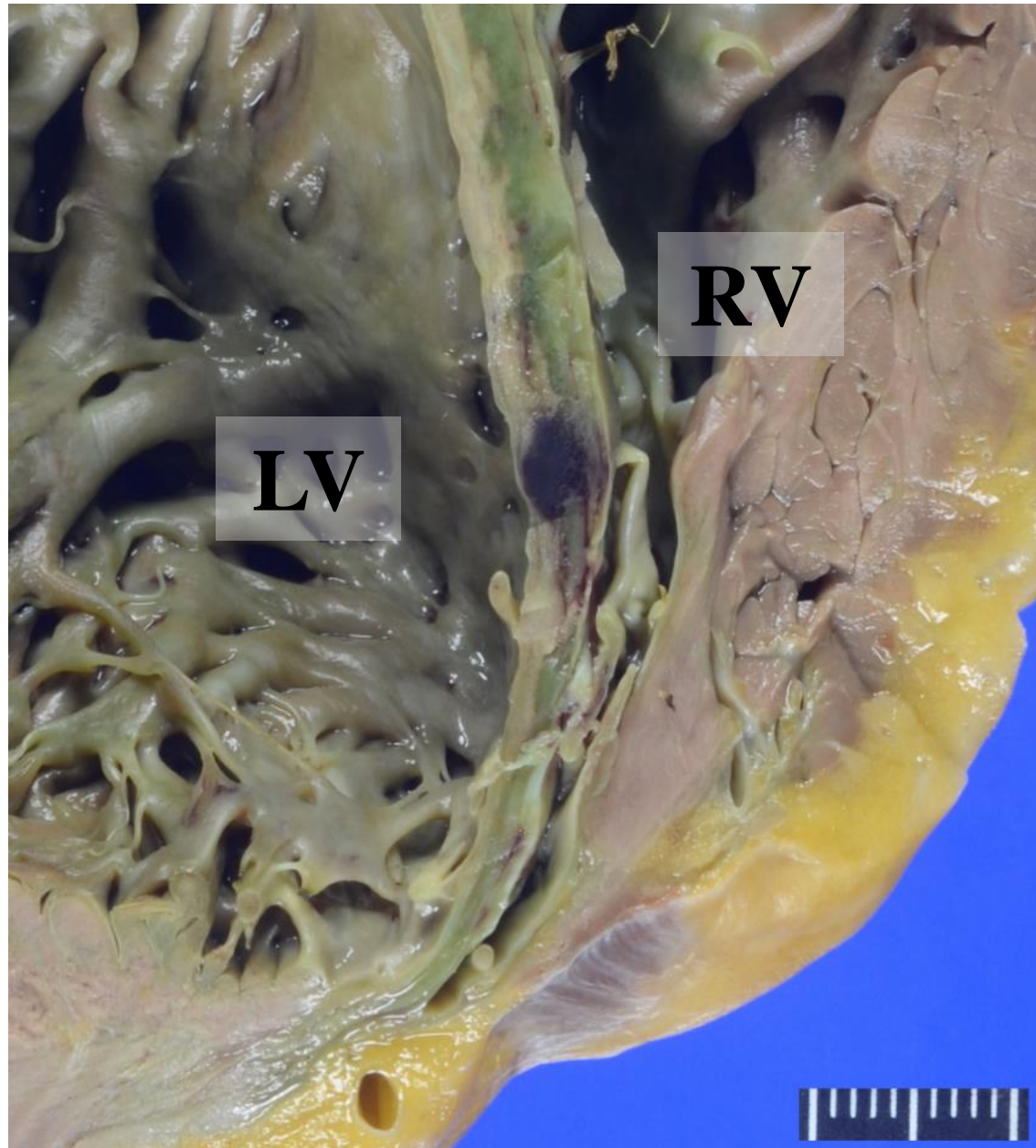

Supplement: Supplementary file 1 [file CCR3-6-1219-s001.pdf]
